# Neurodevelopmental outcome of infants without central nervous system anomalies born to symptomatic RT-PCR ZIKV positive women

**DOI:** 10.1371/journal.pntd.0009854

**Published:** 2022-03-07

**Authors:** Luis Alfonso Díaz-Martínez, Mario Augusto Rojas, Luz Stella Pinilla-García, Carlos Hernán Becerra-Mojica, Luis Alfonso Pérez-Vera, Luz Ángela Gutiérrez-Sánchez, Gustavo Adolfo Contreras-García, Carol Gisela Rueda-Ordoñez, Luis Villar

**Affiliations:** 1 School of Medicine, Health Faculty, Universidad Industrial de Santander, Bucaramanga, Colombia; 2 Colombian Association of Neonatology (ASCON), Colombian Neonatal Research Network (CNRN), Bogotá, Colombia; 3 Maternal-fetal Medicine Unit, Hospital Universitario de Santander, Bucaramanga, Colombia; 4 Neonatal Unit, Hospital Universitario de Santander, Bucaramanga, Colombia; 5 Clínica Materno Infantil San Luis, Bucaramanga, Colombia; 6 Medicina Materno-fetal Integral de Colombia, Bucaramanga, Colombia; 7 ZIKAlliance Consortium, Bucaramanga, Colombia; University of North Carolina at Chapel Hill School of Medicine, UNITED STATES

## Abstract

An epidemic of Zika virus (ZIKV) infection began in Colombia in October 2015. Previous studies have identified a cause-effect relationship between fetal exposure to the ZIKV and the development of microcephaly and other central nervous system (CNS) anomalies with variable degrees of neurodevelopmental delay. Less is known about the neurodevelopmental outcome of infants without CNS anomalies born to symptomatic ZIKV RT-PCR-positive women. We aimed to compare the neurodevelopmental outcome of these infants to a control group of infants without CNS anomalies born to asymptomatic ZIKV RT-PCR negative women who did not seroconvert during pregnancy. Participating infants were categorized according to ZIKV maternal exposure. Women with symptomatology suggestive of ZIKV infection and a positive RT-PCR for ZIKV were categorized as ZIKV-exposed. Maternal controls (ZIKV unexposed) from the same geographic area were subsequently captured during the tail end of the epidemic through a partner project, the *ZIKAlliance*, whose aim was to determine the prevalence of ZIKV in pregnant women. Infant survivors from these two groups of pregnant women had a neurodevelopmental evaluation at 12, 18, and 24 months corrected age (CA). The ZIKV-exposed women were found to be older, had less subsidized health care, had a higher percentage of women in middle-class socioeconomic strata, had higher technical and university education, were less likely to be living with a partner, and had higher rates of pregnancy comorbidity and premature births than ZIKV unexposed women. Compared to infants born to ZIKV unexposed women (*unexposed*), infants born to ZIKV exposed women (*exposed*) were of lower gestational age and required more speech and occupational therapy services. No differences between groups were observed in the proportion of cut-off scores <70 on the Bayley-III Scale at 12, 18, and 24 months for motor, language, and cognitive domains. When a cut-off of <85 was used, a higher percentage of motor and cognitive impairment was observed in *unexposed* infants at 12 and 24 months CA, respectively. Median and IQR score on the Bayley-III scale showed higher scores in favor of *exposed* infants for motor development at 12 and 18 months CA, language at 12 months, and cognitive domain at 12, 18, and 24 months. The adjusted median and IQR compound score of the difference between *exposed and unexposed* was higher in favor of *exposed infants* at 12 to 24 months CA for motor (3.8 [95% CI 1.0 to 6.7]) and cognitive domains (10.6 [95% CI 7.3 to 13.9]). We observed no differences in the language domain (1.9 [95% CI -1.2 to 5.0]). We conclude that infants with no evidence of microcephaly or other CNS anomalies born to ZIKV-exposed women had normal neurodevelopment up to 24 months of CA, supporting an all-or-nothing effect with maternal ZIKV exposure. Long-term follow-up to evaluate school performance is required.

**Clinical Trial Registration:** www.clinicaltrials.gov, NCT02943304.

## Author summary

Previous studies have identified a cause-effect relationship between fetal exposure to the Zika virus (ZIKV) and the development of central nervous system (CNS) anomalies and variable degrees of neurodevelopmental delay. This study aimed to compare the neurodevelopmental outcome of infants without CNS anomalies from two groups. One born to symptomatic ZIKV exposed women and another to asymptomatic ZIKV unexposed women. The ZIKV-exposed women were older, had a higher percentage of women in middle-class socioeconomic strata, had a higher level of education, were more likely to be single, and had higher rates of pregnancy comorbidity and premature births compared to ZIKV unexposed women. Infant survivors from these two groups of pregnant women had a neurodevelopmental evaluation at 12, 18, and 24 months corrected age (CA). Infants born to symptomatic ZIKV exposed women had higher motor and cognitive scores at 12 and 24 months CA, respectively, than infants born to ZIKV unexposed women. We conclude that infants without CNS anomalies born to ZIKV exposed women had normal neurodevelopment up to 24 months of CA than infants born to ZIKV unexposed women, supporting an all-or-nothing effect with maternal ZIKV exposure. School performance evaluation is required.

## Introduction

In 2015, new-onset cases of microcephaly and other central nervous system anomalies were diagnosed in fetuses and infants of women with a previous history of exanthema, conjunctivitis, joint pain, and central nervous system dysfunction from areas of Brazil where the vector, *Aedes* genus, was prevalent. RT-PCR specific for Zika virus (ZIKV) confirmed the cause of this epidemic [1–7]; animal model data subsequently confirmed the neurotropic nature of the virus [8,9]. Confirmed cases of affected fetuses and infants with CNS anomalies were associated with variable degrees of neurodevelopmental impairment [10,11]. Prior to December 2020, many studies evaluated the neurodevelopment of normocephalic infants born to symptomatic women exposed to ZIKV and reported variable degrees of neurodevelopmental impairment. The main limitation of these studies was the absence of a control group for comparison [12–15]. Subsequently, two studies used control groups; the first used a European control group tested with the same motor scale as the exposed population in Brazil, showing significant differences in motor development favoring the control group, but the use of a foreign control group may have biased the results [16]. A second study with an appropriate control group from the same geographic area showed no differences in neurodevelopment between case and control infants. However, their small sample size and a high risk of type II error limited the precision of the estimates of associations of their results [17].

This study aimed to compare the neurodevelopment outcome of a larger population of infants without CNS anomalies, born to symptomatic women who were ZIKV RT-PCR positive, to a control group of infants without CNS anomalies, born to asymptomatic ZIKV RT-PCR and serology negative women from the same region of the ZIKV epidemic in Colombia.

## Materials and methods

### Ethics statement

The ethics and institutional review boards from all participating centers approved this study: Comité Institucional de Ética en Investigación de la Universidad Industrial de Santander, Comité de Ética en Investigación del Hospital Universitario de Santander, Comité de Ética en Investigación Clínica Materno Infantil San Luis, Bucaramanga, Comité de Etica en Investigacion Medicina Materno-fetal Integral de Colombia, Bucaramanga. Formal written consent was obtained from the parent/guardian.

### Study design and participants

This population-based prospective observational study is part of a more extensive study that aimed to better characterize the prevalence of CNS anomalies in fetuses and infants of pregnant women exposed to ZIKV in Bucaramanga, Colombia (www.clinicaltrials.gov, NCT02943304). The study compares the neurodevelopmental outcome of normocephalic infants without CNS anomalies born to ZIKV exposed and unexposed women. ZIKV exposed women had symptoms suggestive of ZIKV infection that was confirmed with RT-PCR. ZIKV unexposed women were asymptomatic, had a negative ZIKV RT-PCR, and had no evidence of seroconversion during pregnancy. Infants born to ZIKV exposed women were categorized as *exposed*. Infants born to ZIKV unexposed women were categorized as *unexposed*. All participating women and infants were recruited from the same geographic area of the epidemic of Zika in Colombia.

Pregnant women who consulted their primary care physician for symptoms suggestive of ZIKV infection were tested with RT-PCR for ZIKV according to the Colombian Ministry of Health guidelines. Symptomatic pregnant women were recruited into the study from four level-III healthcare centers beginning in October 2015. ZIKV RT-PCR testing during the epidemic was centralized at the Instituto Nacional de Salud (INS) in Bogotá, where all biological samples from across the country were processed. Only patients with symptoms suggestive of ZIKV infection were tested with ZIKV RT-PCR due to limitations in diagnostic tests and availability of qualified centers for the test; from a public health perspective, testing of asymptomatic pregnant women was not an option during the acute phase of the epidemic [18].

Given our inability to simultaneously test asymptomatic pregnant women with ZIKV RT-PCR, asymptomatic ZIKV RT-PCR and IgG/IgM negative pregnant women were recruited subsequently at the tail end of the epidemic from the same geographic area through a partnership with the ZIKAlliance Consortium (ZA), a prospective multicenter observational cohort study conducted within the European Commission (EC) Horizon 2020 (H2020)-funded, designed to estimate the absolute and relative risks of congenital abnormalities and adverse outcomes associated with ZIKV infection during pregnancy and to describe the spectrum of abnormalities and adverse pregnancy outcomes associated with ZIKV infection during pregnancy, further characterizing the congenital Zika syndrome [19]; all pregnant women from this cohort were enrolled regardless of symptoms from January 2017 to December 2018 and followed throughout pregnancy and at the time of delivery.

Participating women were enrolled early in pregnancy and followed monthly with serologic testing (IgG and IgM for ZIKV) and RT-PCR for ZIKV in blood and urine. Head ultrasound evaluations for all participating pregnant women were done at least once during the observation period by an obstetrician or a maternal-fetal medicine specialist in 90.2% of cases. Maternal-fetal medicine evaluation included detailed anatomic surveillance for fetal anomalies, independent of the gestational age; 51.4% of these patients had an additional abdominal or vaginal neurosonography performed at the Hospital Universitario de Santander with a pre-established protocol using ISUOG guidelines [20–23]. Microcephaly was characterized as mild or severe. *Mild microcephaly* was defined as a fetal ultrasound measurement of head circumference between -2.99 and -2 standard deviations (SD) from the mean for gestational age; *severe microcephaly* was defined as ≤ -3 SD [22,23]. In addition, placental and infants´ blood/urine samples were collected for ZIKV RT-PCR after birth.

Relevant clinical data on maternal morbidity was captured prospectively, including perinatal and post-partum information such as gestational age at birth, sex, Apgar score at 5 minutes of life, and neonatal anthropometric measurements. Although this study was considered to be of minimal risk to the infant, formal written consent was obtained from the parents or legal guardians for postnatal follow-up and neurodevelopmental evaluation as part of a general consent form that included: screening tests, serologic testing, and imaging of the fetal and neonatal CNS.

### Neurodevelopmental evaluation

Infants with confirmed genetic disorders, STORCH infection, microcephaly, or other CNS anomalies on ultrasound were excluded from all analyses. All *exposed* and *unexposed* infants were referred to the neurodevelopmental follow-up at Hospital Universitario de Santander, Bucaramanga, Colombia. The neurodevelopmental evaluation was done with the Bayley Scale of Infant and Toddler Development, Third Edition (Bayley-III), Spanish edition [24]. An initial visit was planned at six months corrected age (CA) to introduce the mother to the follow-up clinic and its objectives; this evaluation included a general physical exam with anthropometric measurements (head circumference, weight, length). Anthropometric measurements were also performed additionally at the 12, 18, and 24-month visits. All participating infants were then followed up for neurodevelopmental evaluation at 12, 18, and 24 months of CA. The Bayley-III scale was administered individually to evaluate global development key domains: cognitive, language (receptive and expressive), and motor (gross and fine). Cognitive, language, and motor skills were assessed through direct observation of the child in test situations [25]. A physical therapist with expertise in performing the Bayley III (L.S.P.G.) was responsible for all neurodevelopmental evaluations with the support of two trained psychologists and one physical therapist. All evaluators were blinded to the results of RT-PCR for ZIKV, maternal and obstetric history, and to whether they belonged to the *exposed* or *unexposed* group. Due to ethical concerns, referral to physical, speech, or occupational therapy was done at the primary evaluator’s discretion.

To determine differences in neurodevelopment impairment between groups, we used the cut-off scores of <70 and <85 for all domains. We then calculated the median and IQR scores for all domains from the Bayley-III scale between *exposed* and *unexposed* infants. The difference in median IQR was used as a marker for differences in neurodevelopmental outcomes between groups. Finally, expressive and receptive language and fine and gross motor development scores representing the child’s performance in a given sub-score were categorized within a range from 1 to 19, with a mean of 10 and a standard deviation of three.

### Statistical analysis

All relevant and clinical data were collected retrospectively and prospectively from pre-designed data collection tools and stored in a web-based, password-protected electronic research database REDCap [26] with the de-identification capability to protect family and patient sensitive information.

Comparisons between group demographic, clinical characteristics, and Bayley-III scores were made using the Student t or Mann-Whitney test for continuous variables, and Pearson´s χ2 or Fisher exact test for categorical variables. In the language (receptive and expressive) and motor (fine and gross) sub-scores, we calculated the prevalence of patients that scored <7. A significance level of 5% was considered in all analyses.

Finally, crude and adjusted quantile regression models were done to determine median and 95% CI compound score differences on the Bayley-III scores caused by maternal ZIKV exposure while controlling for confounding variables. In these quantile regression models, a cluster variance-covariance matrix analysis was included; the clusters were defined as each infant with follow-up. In this way, the models allowed for intragroup correlation, relaxing the requirement that the observations be independent as it happens in data with repeated observations on individuals. The quantilic model was used because it allows for the unbiased estimation of the difference between the median Bayley-III scores in ZIKV *exposed* and *unexposed* infants, generating a more robust indicator of association than observed with the use of logistic models. [27].

All models were adjusted by socioeconomic level defined by socioeconomic strata (low, middle, high) [28], maternal age, maternal educational level, subsidized healthcare, cohabitation with a partner, and infant´s age and body mass index (BMI) Z-score during each test. BMI was included in the model given the association between nutrition and neurodevelopmental outcome [29]. Similar models were estimated for the language (receptive and expressive) and motor (fine and gross) sub-scores at 12, 18 and, 24 months CA. All analyses were made in Stata/IC 16.1 for Windows (StataCorp LLC, College Station, Texas, USA, 2020).

## Results

**Fig 1:**
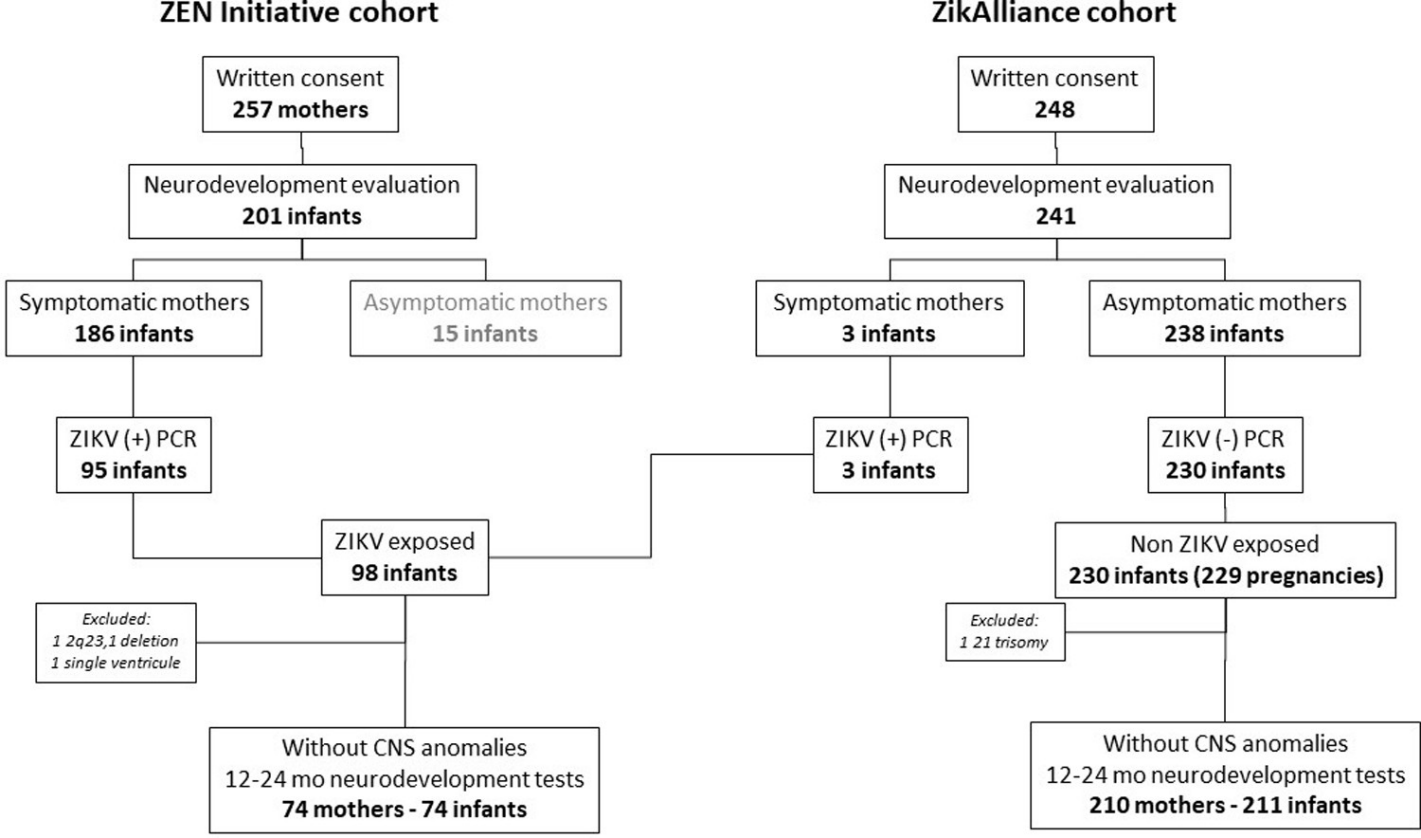
Patient flowchart. Patients were recruited from the same geographic region by two research groups: Zen Initiative and ZikAlliance.

**Fig 2:**
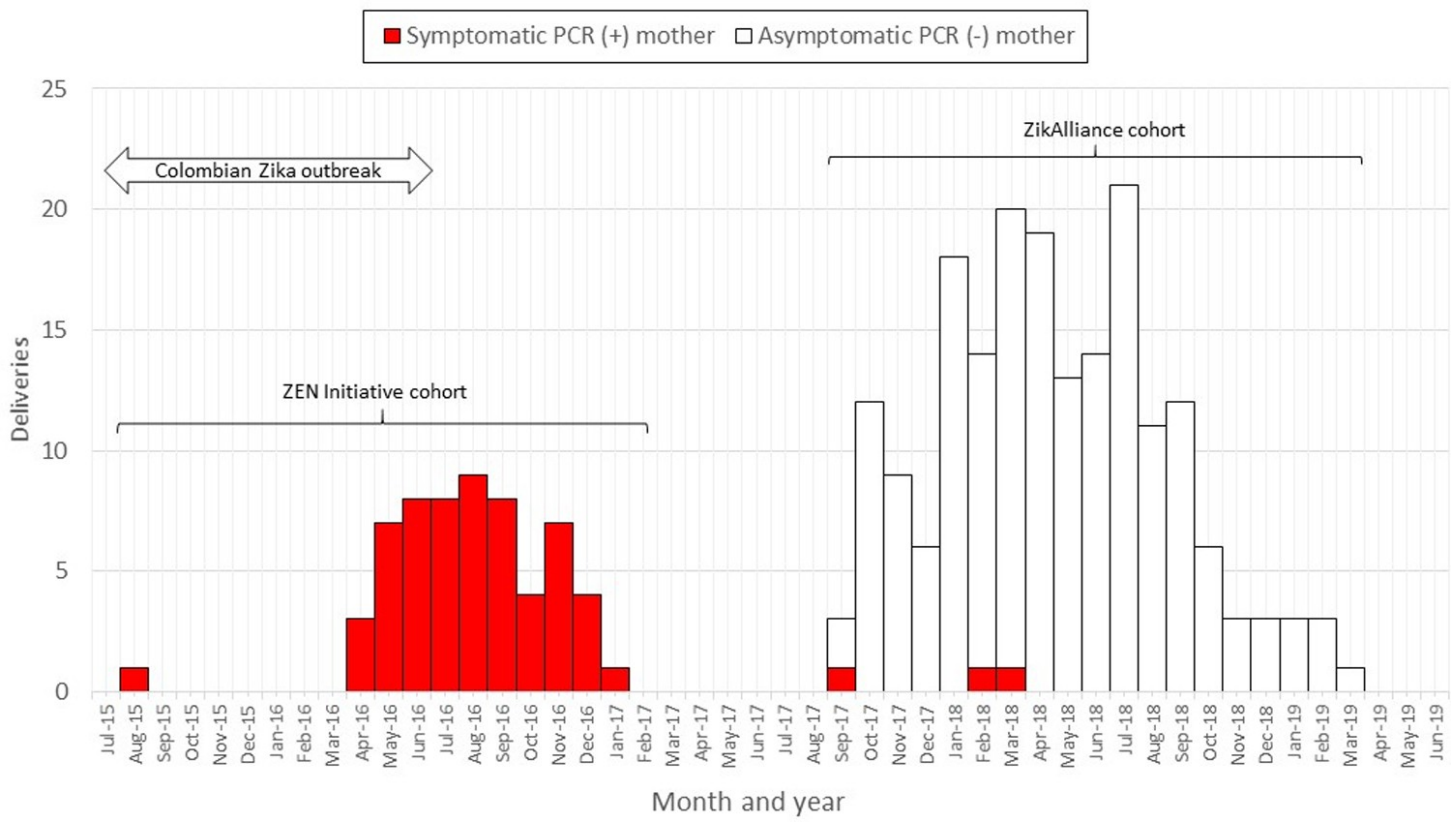
Birthdate month of *exposed* and *unexposed* study infants. Red bars represents the *exposed* infant’s birthday and white bars repesents the *unexposed* infant’s birthday.

A total of 74 symptomatic pregnant women with a positive ZIKV RT-PCR for ZIKV whose infants were born without microcephaly or other CNS anomalies (*exposed*), and 210 asymptomatic pregnant women RT-PCR and serology negative for ZIKV whose infants were born without microcephaly or other CNS anomalies (*unexposed*) were enrolled in this study; three *exposed* infants were recruited from the ZikAlliance cohort (Fig 1). Fig 2 shows the birth month from both *exposed* and *unexposed* infants during the Zika epidemic. Maternal characteristics of ZIKV-exposed and unexposed women are depicted in Table 1. Mothers of the *exposed* infants were older, had a lower percentage of subsidized health care, 70% belonged to low socio-economic strata compared to 88% in ZIKV unexposed women (there were no women from high socioeconomic strata in either group); ZIKV-exposed women were more educated, had higher pregnancy comorbidities, and a higher rate of preterm delivery compared to ZIKV unexposed women. No differences were observed between *exposed* and *unexposed* infants with respect to neonatal characteristics, except for a higher rate of speech and occupational therapy services in the *exposed* group (Table 2).

**Table 1:** Maternal demographic and clinical characteristics of ZIKV exposed and unexposed pregnant women.

Characteristic	Exposed (N = 74)	Unexposed (N = 210)	*p*
Median maternal age (IQR)–yr	25 (22–30)	22 (19–28)	0.001
Subsidized healthcare	46 (62.2%)	201 (95.2%)	<0.001
Low socioeconomic strata	51 (68.9%)	186 (88.6%)	<0.001
Highest educational level			
Elementary	7 (9.5%)	53 (25.2%)	<0.001
High school	35 (47.3%)	130 (61.9%)	
Technical	27 (36.5%)	23 (11.0%)	
University	5 (6.8%)	4 (1.9%)	
Living with a partner	48 (64.9%)	172 (81.9%)	0.003
Previous pregnancies			
None	27 (36.5%)	66 (31.4%)	0.211
1 to 3	45 (60.8%)	126 (60.0%)	
4 or more	2 (2.7%)	18 (8.6%)	
Perinatal infections			
Rubella	0/12 (-)	0/2 (-)	-
Syphilis	1/36 (2.9%)	4/184 (2.2%)	0.824
Citomegalovirus	2/14 (14.3%)	0/2 (-)	0.758
Pregnancy comorbidity			
Urinary tract infections	10/94 (10.6%)	5/201 (2.5%)	0.007
Hypertensive disorders	8/72 (11.1%)	2/209 (1.0%)	<0.001
Diabetes	4/72 (5.6%)	0/204 (-)	0.001
Twin pregnancy	1/74 (1.4%)	1/210 (0.5%)	0.439
Vaginal delivery	37/74 (50.0%)	115/210 (54.8%)	0.284
Median weeks of gestational age at delivery (IQR)	38^2/7^ (370/7–39^4/7^)	39^0/7^ (38^1/7^–40^0/7^)	<0.001
Preterm delivery	11 (14.9%)	11 (5.2%)	0.008

**Table 2:** Demographic and clinical characteristics of *exposed* and *unexposed* infants.

Characteristic	Exposed (N = 74)	Unexposed (N = 211)	p
Neonatal growth			
Small for gestational age	1 (1.4%)	5 (2.4%)	0.352
Appropriate for gestational age	70 (94.5%)	203 (96.2%)	
Large for gestational age	3 (4.1%)	3 (1.4%)	
Female sex	34 (46.0%)	108 (51.2%)	0.438
Apgar Score < 7 at 1 min of life	0 (-)	2 (1.0%)	0.547
Apgar Score < 7 at 5 min of life	0 (-)	0 (-)	-
Patients that received services			
Physical therapy	3 (5.7%)	7 (3.7%)	0.522
Speech therapy	7 (13.2%)	5 (2.6%)	0.002
Occupational therapy	6 (11.3%)	4 (2.1%)	0.003

Table 3 depicts the proportion of infants below the cut-off of <70 and <85 on the Bayley-III Scale of Infant and Toddler Development. No differences were observed between groups for motor, language, and cognitive domains using a cut-off of <70. However, with a cut-off of <85, a higher percentage of motor and cognitive impairment was observed in the *unexposed* group at 12 and 24 months CA, respectively.

**Table 3:** Bayley-III scale domain comparisons below the cut-off of <70 and <85.

Domain and age of assessment	Cut-off <70			Cut-off <85		
	*Exposed*	*Unexposed*	*p*	*Exposed*	*Unexposed*	*p*
***Motor***						
12 mos.	0/48 (-)	3/169 (1.8%)	0.353	2/48 (4.2%)	37/169 (21.9%)	0.002
18 mos.	1/55 (1.8%)	3/128 (2.3%)	0.824	2/55 (3.6%)	12/128 (9.4%)	0.149
24 mos.	0/43 (-)	0/51 (-)	-	1/43 (2.3%)	5/51 (9.8%)	0.146
***Language***						
12 mos.	1/48 (2.1%)	7/169 (4.1%)	0.504	2/48 (4.2%)	52/169 (30.8%)	0.292
18 mos.	8/55 (14.6%)	8/128 (6.3%)	0.069	15/55 (27.3%)	49/128 (38.3%)	0.102
24 mos.	1/43 (2.3%)	0/51 (-)	0.274	12/43 (27.9%)	13/51 (25.5%)	0.487
***Cognitive***						
12 mos.	0/48 (-)	1/169 (0.6%)	0.593	0/48 (-)	5/169 (3.0%)	0.283
18 mos.	0/55 (-)	1/128 (0.8%)	0.511	1/55 (1.8%)	8/128 (6.3%)	0.188
24 mos.	0/43 (-)	0/51 (-)	-	1/43 (2.3%)	8/51 (15.7%)	0.029

Crude comparisons between groups with the median and IQR score on the Bayley-III scale for the three major domains showed a difference in favor of the *exposed case* group at 12 months CA on all domains, at 18 months CA for motor and cognition, and at 24 months CA for cognition alone (Table 4). Throughout the observation period of this study, infants exposed to ZIKV had a lower BMI Z-score (median 0.43, IQR -0.45 to 1.13) than *unexposed* infants (median 0.59, IQR -0.19 to 1.40; p = 0.026), justifying its inclusion in the adjusted models. Adjusted quantile regression models (Table 5) comparing *exposed* and *un*exposed infants showed significant differences between groups at 12 months CA for motor, and at 12, 18, and 24 months for cognition. No differences were observed for the language domain.

**Table 4:** Median and IQR score on the Bayley-III scale domains between *exposed* and *unexposed* infants.

Domain and age of assessment	*Exposed*		*Unexposed*		*p*
	n	*Median (IQR)*	n	*Median (IQR)*	
***Motor***					
12 mos.	48	110 (94–118)	170	92.5 (85–100)	<0.001
18 mos.	55	103 (97–107)	128	100 (94–103)	0.003
24 mos.	43	94 (91–100)	51	94 (91–100)	0.944
***Language***					
12 mos.	48	97 (91–104.5)	170	89 (83–97)	<0.001
18 mos.	55	89 (79–97)	128	89 (79–97)	0.857
24 mo.	43	91 (79–97)	51	91 (83–97)	0.618
***Cognitive***					
12 mos.	48	120 (110–125)	170	100 (95–105)	<0.001
18 mos.	55	105 (100–115)	128	95 (95–100)	<0.001
24 mos.	43	100 (90–105)	51	95 (85–95)	<0.001

**Table 5:** Crude and adjusted median and 95% CI compound score differences between *exposed* and *unexposed* infants on the Bayley-III scale domains.

Domain and age of assessment	Crude median difference	Adjusted median difference*
***Motor***		
12 mos.	12.0 (2.1 to 212.9)	12.8 (3.4 to 22.3)
18 mos.	3.0 (0.8 to 5.2)	2.2 (-2.0 to 6.3)
24 mos.	0.0 (-5.3 to 5.3)	-0.7 (-5.8 to 4.5)
12 to 24 mo.	4.0 (1.2 to 6.9)	3.8 (1.0 to 6.7)
***Language***		
12 mos.	8.0 (4.2 to 11.8)	4.8 (-0.2 to 9.9)
18 mos.	0.0 (-6.3 to 6.3)	0.3 (-4.8 to 5.5)
24 mos.	-2.0 (-8.1 to 4.1)	-4.5 (-13.3 to 4.4)
12 to 24 mos.	2.0 (-0.8 to 4.8)	1.9 (-1.2 to 5.0)
***Cognitive***		
12 mos.	15.0 (8.8 to 21.2)	14.3 (7.8 to 20.9)
18 mos.	10.0 (4.7 to 15.3)	8.4 (2.6 to 14.2)
24 mos.	5.0 (-1.2 to 11.2)	11.1 (3.0 to 19.2)
12 to 24 mos.	11.2 (7.7 to 14.7)	10.6 (7.3 to 13.9)

*Adjusted by socioeconomic strata, maternal education level, social security maternal age, mother living with a partner, and infant´s age and body mass index Z-score during each test.

The prevalence of <7 score in fine motor skills was higher in *exposed* than *unexposed* infants at 12 months CA, but not at 18 or 24 months; the same occurred with expressive language (Table 6); with respect to receptive language, a similar situation was observed at 12 and 18 months CA, but not at 24 months CA. There were no differences in the prevalence of <7 scored in gross motor skills. Finally, the adjusted median sub-score differences showed similar conclusions in fine and gross motor skills at 12 and 18 months CA, and in expressive language at 12 months CA.

**Table 6:** Bayley-III sub-scale comparisons below the cut-off of <7 for motor and language domains.

Sub-scale and age of assessment	*Exposed*	*Unexposed*	*p*	Adjusted median and 95% CI sub-score differences*
***Fine motor***				
12 mos.	0/48 (-)	10/169 (5.9%)	0.077	5.94 (4.30 to 7.58)
18 mos.	2/55 (3.6%)	8/128 (6.3%)	0.376	0.86 (0.27 to 1.45)
24 mos.	1/43 (2.3%)	1/51 (2.0%)	0.708	0.18 (-0.91 to 1.28)
***Gross motor***				
12 mos.	7/48 (14.6%)	40/169 (23.7%)	0.123	0.36 (-0.94 to 1.67)
18 mos.	4/55 (1.8%)	18/128 (14.1%)	0.147	1.07 (0.30 to 1.84)
24 mos.	6/43 (14.0%)	7/51 (13.7%)	0.603	-0.46 (-1.33 to 0.39)
***Expressive language***				
12 mos.	4/48 (8.3%)	67/169 (39.6%)	<0.001	2.56 (1.74 to 3.39)
18 mos.	14/55 (25.5%)	52/128 (40.6%)	0.124	1.30 (-0.07 to 2.67)
24 mos.	12/43 (27.9%)	16/51 (31.4%)	0.186	-0.49 (-2.18 to 1.22)
***Receptive language***				
12 mos.	2/48 (4.2%)	30/169 (17.8%)	0.011	1.50 (-0.46 to 3.47)
18 mos.	12/55 (21.8%)	16/128 (12.5%)	0.086	-0.11 (-1.75 to 1.53)
24 mos.	4/43 (9.3%)	1/51 (2.0%)	0.132	-0.68 (-1.76 to 0.41)

*Adjusted by socioeconomic strata, maternal education level, social security, maternal age, mother living with a partner, and infant´s age and body mass index Z score during each sub-test.

## Discussion

This prospective study shows no evidence of neurodevelopmental impairment in *exposed* infants compared to *unexposed* infants. This result suggests that maternal infection with ZIKV, if not associated with fetal or neonatal microcephaly or other CNS anomaly, has in itself no effect on neurodevelopment up to twenty-four months CA, supporting an all-or nothing-effect. Explanations for why infants of ZIKV exposed women had higher cognition scores at 24 months CA than *unexposed* infants, even when both *exposed* and *unexposed* infants had their Bayley III scores performed by the same-blinded evaluators, may be twofold. First, knowing the potential risk of neonatal neurodevelopmental compromise with Zika infection, more educated ZIKV exposed mothers may have been more prone to stimulating their infants than ZIKV unexposed mothers. The higher rate of occupational and speech therapy in *exposed* infants most likely was associated with a higher rate of premature births in this group. Second, the ZIKV unexposed women may not have been aware of the need to make any additional efforts to stimulate their more mature infants who were thought to be at low risk for ZIKV infection given their mother’s asymptomatic status and enrollment towards the end of the epidemic. Lower cognition scores in *unexposed* infants may be explained by socioeconomic status and maternal education associated with parental stimulation deficits. However, the adjusted multivariate models show an independent effect of ZIKV exposure on poor neurodevelopmental outcomes that may have been countered by placental protective mechanisms avoiding the transfer of ZIKV to the fetal brain and increased parental stimulation in the *exposed* group.

Previous studies evaluating the neurodevelopmental outcome of normocephalic infants born to ZIKV exposed women found significant anomalies in neurodevelopment. However, these studies did not include an appropriate control group for comparison [12–15, 30–32] or had a high risk of selection bias [33–36]. A study by Andrade et al. [32] used a similar methodology to study *exposed* infants observing a higher rate of receptive language delay, but the absence of a control group limited the study; our study did not find any differences in receptive or expressive language between *exposed* and *un*exposed infants using the same Bayley III score. Aguilar-Ticona et al. [37] included an appropriate control group showing mild cognitive delay and auditory behavioral abnormalities in infants of ZIKV exposed women but was limited by small sample size and wide ranges in confidence intervals of relative risks describing these associations. Gerzson et al. [17] used an appropriate control group and although limited by small sample size, did not find any difference between normocephalic *exposed* and *unexposed* infants in neurodevelopmental outcome between 18 and 29 months CA. A prospective cohort study by Grant et al. [36] studied symptomatic pregnant women with ZIKV RT-PCR or serology to determine the exposure status of infants. They evaluated neurodevelopmental outcome at 24 months with the ASQ3, a screening tool for neurodevelopmental impairment, and found no differences between *exposed* infants and infants of symptomatic RT-PCR and serology negative women (*controls*).

Different from the previous study, we used infants of asymptomatic ZIKV RT-PCR and serology negative women to minimize misclassification bias; also, compared to the Bayley III scale that was used in our study, the ASQ3 has shown significant variability in sensitivity and specificity as a screening tool for neurodevelopmental anomalies but improves as the infant advances in age. Our results support the findings of Gerzson et al. [17] and Grant et al. [36] and highlight the importance of having an adequate unexposed control group to determine differences in outcome incidence as a critical element of causality and to reduce selection bias risk in observational studies [38].

Factors related to the ZIKV, placental maternal-fetal barrier characteristics, fetal immune system, and time of infection during pregnancy may individually or collectively play an essential role in the frequency and severity of fetal compromise. The importance of the first-trimester infection and its relation to the CNS anomalies and severe neurodevelopmental delay underscores the role of placental maternal-fetal barrier maturity in fetal pathogenesis with ZIKV infection, among other plausible explanations [39–43].

A limitation of this study was the difference in enrollment time between *exposed* and *unexposed* infants; enrollment of ZIKV unexposed pregnant women occurred at the tail end of the epidemic, which may partially explain the differences in socio-economic and education levels between groups. At the beginning of the epidemic, testing with RT-PCR for ZIKV was focused on symptomatic mothers due to our lack of knowledge about asymptomatic infections and the limited availability of testing kits and qualified centers for the test. We addressed the need to have an appropriate control group by partnering with ZikAlliance; through mutual collaboration, we identified and recruited asymptomatic pregnant women from the same geographical area. These women were followed prospectively throughout their pregnancy and confirmed to have negative RT-PCR and serologies for ZIKV. Another limitation is the Bayley-III scale and its ability to predict school-age outcomes [44]. Given this limitation, we made an effort to minimize other sources of evaluation bias by having the same-blinded evaluators perform the Bayley-III scale for *exposed* and *unexposed* infant groups.

False-negative results with RT-PCR for ZIKV have been a limitation for all studies published to date. However, asymptomatic pregnant women were tested both at the beginning and the end of their pregnancies to categorize their infants as *unexposed* and minimize misclassification bias [45]. Fetal ultrasound confirmed the absence of CNS anomalies. The monthly maternal follow up of this group with ZIKV RT-PCR, serologies, or both also minimized the risk of misclassification bias.

In summary, our study showed that *exposed* infants with no evidence of microcephaly or CNS anomalies had normal neurodevelopmental outcomes up until 24 months CA, supporting an all-or-nothing effect of maternal ZIKV exposure. Follow-up of *exposed* and *unexposed* infants at age five is recommended to better characterize school performance. This study will help physicians, and health care workers determine the risk of fetal compromise to better inform pregnant women and support public health experts with the planning and distribution of health care resources.

## Supporting information

S1 Text Data and Dictionary. (XLS)

## References

[1] Ladhani SN, O’Connor C, Kirkbride H, Brooks T, Morgan D. Outbreak of Zika virus disease in the Americas and the association with microcephaly, congenital malformations and Guillain–Barré syndrome. Arch Dis Child. 2016;101: 600–602. doi: 10.1136/archdischild-2016-310590 26998633 PMC4941169

[2] Collins MH, Zepeda O, Blette B, Jadi R, Morales M, Pérez R, et al. Serologic surveillance of maternal Zika infection in a prospective cohort in Leon, Nicaragua during the peak of the Zika epidemic. PLoS One. 2020;15: e0230692. doi: 10.1371/journal.pone.0230692 32243482 PMC7122769

[3] Heukelbach J, Werneck GL. Surveillance of Zika virus infection and microcephaly in Brazil. Lancet. 2016;388: 846–847. doi: 10.1016/S0140-6736(16)30931-X 27372396

[4] Duffy MR, Chen TH, Hancock WT, Powers AM, Kool JL, Lanciotti RS, et al. Zika virus outbreak on Yap Island, Federated States of Micronesia. N Engl J Med. 2009;360: 2536–2543. doi: 10.1056/NEJMoa0805715 19516034

[5] Ioos S, Mallet HP, Leparc Goffart I, Gauthier V, Cardoso T, Herida M. Current Zika virus epidemiology and recent epidemics. Med Mal Infect. 2014;44: 302–307. doi: 10.1016/j.medmal.2014.04.008 25001879

[6] Cauchemez S, Besnard M, Bompard P, Dub T, Guillemette-Artur P, Eyrolle-Guignot D, et al. Association between Zika virus and microcephaly in French Polynesia, 2013–2015: A retrospective study. Lancet. 2016;71: 512–514. doi: 10.1097/01.ogx.0000491260.72445.aa PMC4909533 26993883

[7] Rasmussen SA, Jamieson DJ, Honein MA, Petersen LR. Zika virus and birth defects—Reviewing the evidence for causality. N Engl J Med. 2016;374: 1981–1987. doi: 10.1056/NEJMsr1604338 27074377

[8] Hickman HD, Pierson TC. Zika in the brain: New models shed light on viral infection. Trends Mol Med. 2016;22: 639–641. doi: 10.1016/j.molmed.2016.06.004 27345865 PMC4990132

[9] Cugola FR, Fernandez IR, Russo FB, Freitas BC, Dias JLM, Guimaraes KP, et al. The Brazilian Zika virus strain causes birth defects in experimental models. Nature. 2016;534: 267–271. doi: 10.1038/nature18296 27279226 PMC4902174

[10] Carvalho A, Brites C, Mochida G, Ventura P, Fernandes A, Lage ML, et al. Clinical and neurodevelopmental features in children with cerebral palsy and probable congenital Zika. Brian Dev. 2019;41: 587–594. doi: 10.1016/j.braindev.2019.03.005 30914212

[11] Mendes AKT, Ribeiro MRC, Lamy-Filho F, Amaral GA, Borges MCR, Costa LC, et al. Congenital Zika syndrome: association between the gestational trimester of maternal infection, severity of brain computed tomography findings and microcephaly at birth. Rev Inst Med Trop Sao Paulo. 2020;62: 1–8. doi: 10.1590/s1678-9946202062056 32844907 PMC7447234

[12] Mulkey SB, Arroyave-Wessel M, Peyton C, Bulas DI, Fourzali Y, Jiang J, et al. Neurodevelopmental Abnormalities in Children with in Utero Zika Virus Exposure Without Congenital Zika Syndrome. JAMA Pediatr. 2020;174: 269–276. doi: 10.1001/jamapediatrics.2019.5204 31904798 PMC6990858

[13] Peçanha PM, Gomes Junior SC, Pone SM, Pone MV da S, Vasconcelos Z, Zin A, et al. Neurodevelopment of children exposed intra-uterus by Zika virus: A case series. PLoS One. 2020;15: e0229434. doi: 10.1371/journal.pone.0229434 32109947 PMC7048286

[14] Nielsen-Saines K, Brasil P, Kerin T, Vasconcelos Z, Gabaglia CR, Damasceno L, et al. Delayed childhood neurodevelopment and neurosensory alterations in the second year of life in a prospective cohort of ZIKV-exposed children. Nat Med. 2019;25: 1213–1217. doi: 10.1038/s41591-019-0496-1 31285631 PMC6689256

[15] Faiçal AV, De Oliveira JC, Oliveira JVV, De Almeida BL, Agra IA, Alcantara LCJ, et al. Neurodevelopmental delay in normocephalic children with in utero exposure to Zika virus. BMJ Paediatr Open. 2019;3: 9–11. doi: 10.1136/bmjpo-2019-000486 31338431 PMC6613842

[16] Einspieler C, Utsch F, Brasil P, Panvequio Aizawa CY, Peyton C, Hydee Hasue R, et al. Association of infants exposed to prenatal Zika virus infection with their clinical, neurologic, and developmental status evaluated via the General Movement Assessment Tool. JAMA Netw open. 2019;2: e187235. doi: 10.1001/jamanetworkopen.2018.7235 30657537 PMC6431234

[17] Gerzson LR, de Almeida CS, Silva JH da, Feitosa MMA, de Oliveira LN, Schuler-Faccini L. Neurodevelopment of nonmicrocephalic children, after 18 months of life, exposed prenatally to Zika virus. J Child Neurol. 2020;35: 278–282. doi: 10.1177/0883073819892128 31878830

[18] Ospina ML, Tong VT, González M, Valencia D, Mercado M, Gilboa SM, et al. Zika virus disease and pregnancy outcomes in Colombia. N Engl J Med. 2020;383: 537–545. doi: 10.1056/NEJMoa1911023 32757522 PMC7480270

[19] Avelino-Silva VI, Mayaud P, Tami A, Miranda MC, Rosenberger KD, Alexander N, et al. Study protocol for the multicentre cohorts of Zika virus infection in pregnant women, infants, and acute clinical cases in Latin America and the Caribbean: The ZIKAlliance consortium. BMC Infect Dis. 2019;19: 1–14. doi: 10.1186/s12879-018-3567-x 31878895 PMC6933915

[20] Salomon LJ, Alfirevic Z, Berghella V, Bilardo C, Hernandez-Andrade E, Johnsen SL, et al. Practice guidelines for performance of the routine mid-trimester fetal ultrasound scan. Ultrasound Obstet Gynecol. 2011;37(1):116–26. doi: 10.1002/uog.8831 20842655

[21] Paladini D, Malinger G, Monteagudo A, Pilu G, Timor-Tritsch I, Toi A. Sonographic examination of the fetal central nervous system: Guidelines for performing the “basic examination” and the “fetal neurosonogram.” Ultrasound Obstet Gynecol. 2007;29(1):109–16. doi: 10.1002/uog.3909 17200992

[22] Papageorghiou AT, Thilaganathan B, Bilardo CM, Ngu A, Malinger G, Herrera M, et al. ISUOG Interim Guidance on ultrasound for Zika virus infection in pregnancy: Information for healthcare professionals. Ultrasound Obstet Gynecol. 2016;47(4):530–2. doi: 10.1002/uog.15896 26969966

[23] Gutierrez-Sanchez LA, Becerra-Mojica CH, Rojas MA, Diaz-Martinez LA, Perez-Vera LA, Contreras-Garcia GA et al. Fetal central nervous system anomalies according to RT-PCR and trimester of infection with Zika virus: A prospective Cohort study. Acta Obstet Gynecol Scand. 2021;00:1–11. doi: 10.1111/aogs.14301 34904224 PMC9564424

[24] Bayley N. Escalas Bayley de desarrollo infantil-III. Manual de aplicación. Madrid: PsycoCorp; 2005.

[25] Johnson S, Moore T, Marlow N. Using the Bayley-III to assess neurodevelopmental delay: Which cut-off should be used? Pediatr Res. 2014;75: 670–674. doi: 10.1038/pr.2014.10 24492622

[26] Harris PA, Taylor R, Thielke R, Payne J, Gonzalez N, Conde JG. Research Electronic Data Capture (REDCap)—A metadata-driven methodology and workflow process for providing translational research informatics support Nathaniel. J Biomed Inf. 2009;42: 377–381. doi: 10.1016/j.jbi.2008.08.010.Research PMC2700030 18929686

[27] Rabe-Hesketh S, Skrondal A. Multilevel and longitudinal modeling using Stata. Volumen II: Categorical responses, counts, and survival. 3rd ed. College Station, Texas: Stata Press; 2012.

[28] Departamento Administrativo Nacional de Estadística. Metodología de estratificación socioeconómica urbana para servicios públicos domiciliarios. 2nd ed. Bogotá, Colombia: Departamento Administrativo Nacional de Estadística; 2015. Available: https://www.dane.gov.co/files/geoestadistica/estratificacion/EnfoqueConceptual.pdf

[29] Georgieff MK, Ramel SE, Cusik SE. Nutritional influences on brain development. Acta Paediatr. 2018;107: 1310–1321. doi: 10.1111/apa.14287 29468731 PMC6045434

[30] Aizawa CYP, Caron DMR, Souza CB de, Kozima PFA, Damasceno L, Einspieler C, et al. Neurodevelopment in the third year of life in children with antenatal ZIKV-exposure. Rev Saude Publica. 2021;55: 15. doi: 10.11606/s1518-8787.2021055002798 33909869 PMC8032323

[31] Cardona-Ospina JA, Zapata MF, Grajales M, Arias MA, Grajales J, Bedoya-Rendón HD, et al. Physical Growth and Neurodevelopment of a Cohort of Children after 3.5 Years of Follow-up from Mothers with Zika Infection during Pregnancy-Third Report of the ZIKERNCOL Study. J Trop Pediatr. 2021;67: 1–10. doi: 10.1093/tropej/fmab032 34037794

[32] Andrade LM, Baker Meio MD, Gomes SC, Souza JP, Figueiredo MR, Costa RP, et al. Language delay was associated with a smaller head circumference at birth in asymptomatic infants prenatally exposed to the Zika virus. Acta Paediatr Int J Paediatr. 2021;110: 2375–2381. doi: 10.1111/apa.15878 33872416 PMC8286322

[33] Gazeta RE, Bertozzi APAP, Dezena R de C de AB, Silva ACB, Fajardo TCG, Catalan DT, et al. Three-year clinical follow-up of children intrauterine exposed to zika virus. Viruses. 2021;13: 1–15. doi: 10.3390/v13030523 33810110 PMC8005078

[34] Hcini N, Kugbe Y, Rafalimanana ZHL, Lambert V, Mathieu M, Carles G, et al. Association between confirmed congenital Zika infection at birth and outcomes up to 3 years of life. Nat Commun. 2021;12: 3270. doi: 10.1038/s41467-021-23468-3 34075035 PMC8169933

[35] Stringer EM, Martínez E, Blette B, Toval Ruiz CE, Boivin M, Zepeda O, et al. Neurodevelopmental outcomes of children following in utero exposure to Zika in Nicaragua. Clin Infect Dis. 2021;72: E146–E153. doi: 10.1093/cid/ciaa1833 33515459 PMC7935385

[36] Grant R, Fléchelles O, Tressières B, Dialo M, Elenga N, Mediamolle N, et al. In utero Zika virus exposure and neurodevelopment at 24 months in toddlers normocephalic at birth: a cohort study. BMC Med. 2021;19: 12. doi: 10.1186/s12916-020-01888-0 33472606 PMC7819189

[37] Aguilar-Ticona JP, Nery N, Ladines-Lim JB, Gambrah C, Sacramento G, de Paula Freitas B, et al. Developmental outcomes in children exposed to zika virus in utero from a brazilian urban slum cohort study. PLoS Negl Trop Dis. 2021;15: e0009162. doi: 10.1371/journal.pntd.0009162 33544730 PMC7891708

[38] Reveiz L, Haby MM, Martínez-Vega R, Pinzón-Flores CE, Elias V, Smith E, et al. Risk of bias and confounding of observational studies of Zika virus infection: A scoping review of research protocols. PLoS One. 2017;12: e0180220. doi: 10.1371/journal.pone.0180220 28686621 PMC5501456

[39] Pass RF, Stagno S, Myres GL, Alford CA. Outcome of symptomatic congenital cytomegalovirus infection: results of long-term longitudinal follow-up. Pediatrics. 1980;66: 758–762. 6159568

[40] Gutiérrez-Sánchez LA, Sandoval-Martínez DK, Díaz-Martínez LA, Becerra-Mojica CH. Zika virus infection: A correlation between prenatal ultrasonographic and postmortem neuropathologic changes. Neuropathology. 2019;39: 434–440. doi: 10.1111/neup.12603 31710135

[41] Raper J, Kovacs-Balint Z, Mavigner M, Gumber S, Burke MW, Habib J, et al. Long-term alterations in brain and behavior after postnatal Zika virus infection in infant macaques. Nat Commun. 2020;11: 1–12. doi: 10.1038/s41467-019-13993-7 32439858 PMC7242369

[42] Barbeito-Andrés J, Pezzuto P, Higa LM, Dias AA, Vasconcelos JM, Santos TMP, et al. Congenital Zika syndrome is associated with maternal protein malnutrition. Sci Adv. 2020;6: eaaw6284. doi: 10.1126/sciadv.aaw6284 31950075 PMC6954064

[43] Zhang W, Tan YW, Yam WK, Tu H, Qiu L, Tan EK, et al. In utero infection of Zika virus leads to abnormal central nervous system development in mice. Sci Rep. 2019;9: 1–12. doi: 10.1038/s41598-018-37186-2 31086212 PMC6513999

[44] O’Shea TM, Joseph RM, Allred EN, Taylor HG, Leviton A, Heeren T et al. ELGAN Study Investigators. Accuracy of the Bayley-II mental development index at 2 years as a predictor of cognitive impairment at school age among children born extremely preterm. J Perinatol. 2018;38: 908–916. doi: 10.1038/s41372-017-0020-8 29808002 PMC6070417

[45] Paiva MHS, Guedes DRD, Leal WS, Ayres CFJ. Sensitivity of RT-PCR method in samples shown to be positive for zika virus by RT-qPCR in vector competence studies. Genet Mol Biol. 2017;40: 597–599. doi: 10.1590/1678-4685-GMB-2016-0312 28534930 PMC5596374

